# Unraveling the links between circulating bioactive factors and epilepsy: A bidirectional Mendelian randomization study

**DOI:** 10.1097/MD.0000000000038256

**Published:** 2024-05-31

**Authors:** Wencai Wang, Luyao Ma, Menghao Liu, Yongqiang Zhao, Wei Ye, Xianfeng Li

**Affiliations:** a The Second Affiliated Hospital of Harbin Medical University, Harbin, People’s Republic of China.

**Keywords:** bioactive factors, biomarkers, focal epilepsy, generalized epilepsy, inflammatory cytokines, Mendelian randomization

## Abstract

Epidemiological research has shown that a variety of circulating bioactive factors are associated with epilepsy, including macrophage colony-stimulating factor, interleukin-1β, and tumor necrosis factor-α. To further investigate the associations between epilepsy and 41 inflammatory cytokines, this Mendelian randomization was performed. This study presents genome-wide association study summary data on 41 inflammatory cytokines and epilepsy. Epilepsy incorporates generalized and focal epilepsy. A two-sample Mendelian randomization method was used. In order to analyze causal relationships between exposures and outcomes, the inverse variance-weighted method was mainly used. The findings suggested that increased levels of interleukin-1 receptor antagonists and interleukin-5 may be significantly associated with increased risks of focal epilepsy (beta: 0.080, *P* = .043; beta: 0.083, *P* = .015). In addition, regulated upon activation normal T cell expressed and secreted factor and Macrophage colony-stimulating factor may be significantly associated with generalized epilepsy (beta: 0.110, *P* = .042; beta: –0.114, *P* = .024). Furthermore, inflammatory cytokines such as interleukin-10, interleukin-1β, interleukin-1Ra, interleukin-7, tumor necrosis factor-α, and interferon-γ may be identified as the result of focal epilepsy (beta: 0.152, *P* = .031; beta: 0.214, *P* = .037; beta: 0.214, *P* = .047; beta: 0.222, *P* = .031; beta: 0.224, *P* = .025; beta: 0.161, *P* = .018). This study suggests that interleukin-5 and interleukin-1 receptor antagonists are potentially correlated factors with focal epilepsy etiology, macrophage colony-stimulating factor and regulated upon activation normal T cell expressed and secreted factor are potentially correlated factors with generalized epilepsy etiology, while several inflammatory cytokines possibly contribute to focal epilepsy development downstream.

## 1. Introduction

Epilepsy is one of the most widespread chronic neurological diseases. Epidemiological studies show that the prevalence in the population is approximately 1.0% to 2.0% and can occur at any age.^[1]^ According to the International League Against Epilepsy (ILAE) classification criteria, epilepsy is clinically divided into focal and generalized epilepsy.^[2]^ Although the specific etiology and pathophysiology of focal epilepsy are still unknown, the critical impact of inflammation in the occurrence and recurrence of the disease has been confirmed by several previous studies.^[3–5]^ The inflammatory state observed in patients with epilepsy is characterized by overactive and inappropriate immune effector cell functions and elevated levels of specific inflammatory cytokines, including interleukin (IL)-6, tumor necrosis factor (TNF)-α, and interleukin (IL)-1β.^[6]^ Ultimately, this leads to neuronal damage and abnormal discharges. In addition, several other bioactive factors have been described to be strongly associated with epilepsy, such as macrophage colony-stimulating factor (MCSF),^[7]^ interleukin (IL)-5,^[8,9]^ and interleukin-1 receptor antagonists (IL-1Ra).^[10]^ However, it remains controversial whether inflammation causes epilepsy or is due to the use of medications, disease development, and subsequent infection after the onset of epilepsy. Although some observational research has tried to clarify the links between epilepsy and bioactive factors, results may be confounded by unforeseen confounders or reversed causality, which makes it challenging to establish definitive causal relationships.

Mendelian randomization (MR), a recognized analytical technique, employs genetic variation in nonexperimental data to deduce the causative impact of exposures upon outcomes.^[11]^ Like randomized controlled trials, MR analyses are more robust in reversing causality and confounders because genotypes are assigned arbitrarily during gamete fusion.^[12]^ Increasing the usability and effectiveness of the test, two-sample MR analysis permits investigators to examine the relationships between exposures and outcomes in 2 populations.^[13]^ This study extracted reliable genetic variations from 41 bioactive factors genome-wide association study (GWAS) summaries to examine their links to epilepsy. It also investigated the direction of causality by reversal of exposures and outcomes.

## 2. Methods

### 2.1. MR analysis

**Figure 1.**
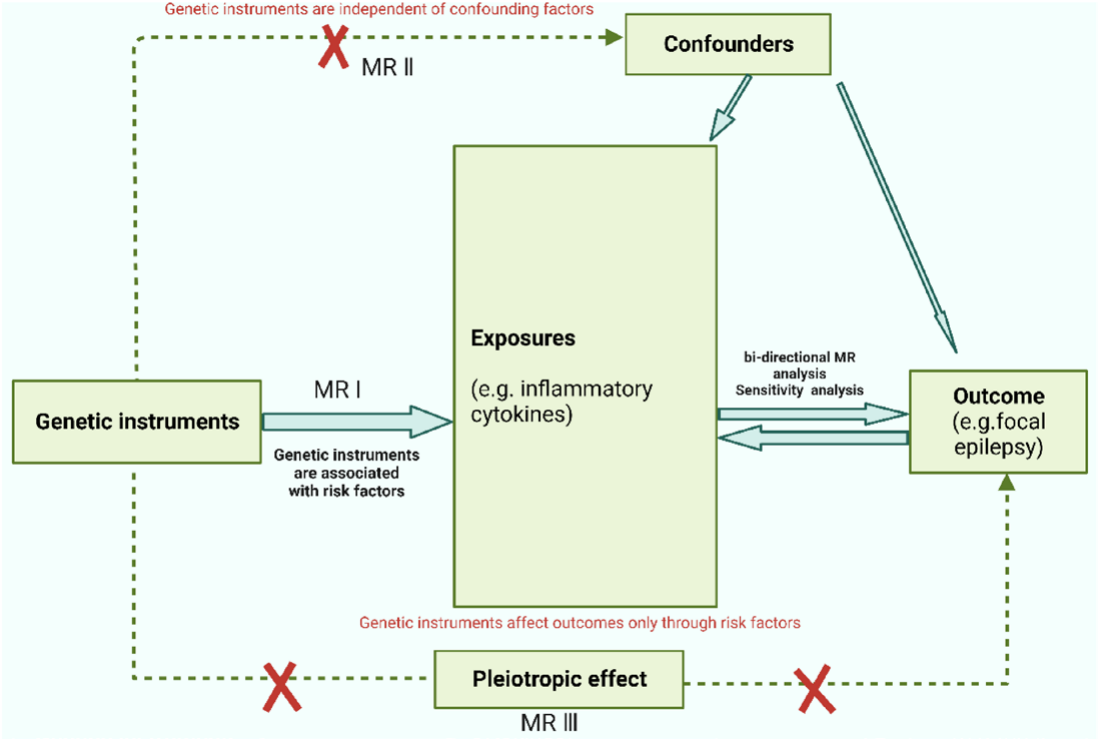
Schematic of the study design in this bidirectional Mendelian randomization (MR) analysis.

For this analysis, we utilized published summary statistics from GWAS. We have followed the STROBE-MR checklist for conducting MR study and writing the article.^[14]^ Relevance, independence, and constraint are the 3 main assumptions of MR analysis.^[15]^ (1) The genetic variations that have been selected are related to the risk factors (relevant); (2) there is no confounding between risk factors and outcomes (independent); (3) the outcomes cannot occur otherwise than via the risk factors of interest (constrained) (Fig. 1).

### 2.2. Data source

Two datasets from publicly available GWAS summary data were used in this MR analysis. For bioactive factors, the database came from a study examining the association of genomic variants with 41 growth factors and cytokines in 8293 people from Finland^[16,17]^ (Table 1). This data set contains pooled meta-analysis statistics from inflammatory cytokine GWAS conducted in 3 Finnish cohorts (Young Finns Study and FINRISK, 1997 and 2002). The average age of those participating in the Young Finns Study was 37, while the average age of those participating in the FINRISK study was 60. This data set is the published GWAS data reported by Ahola-Olli et al, published in the American Journal of Human Genetics, reanalyzed in 2020 by Laura Corbin, Nicholas Timpson et al, and published in a public database by the University of Bristol, which can be publicly accessed at https://doi.org/10.5523/bris.3g3i5smgghp0s2uvm1doflkx9x.

**Table 1:** Sample size for each cytokine analyzed in this study acquired from the GWAS.

Abbreviation	Cytokines	Sample size	Number
*Chemokines*			
CTACK	Cutaneous T-cell attracting (CCL27)	3631	GCST004420
Eotaxin	Eotaxin (CCL11)	8153	GCST004460
GROa	Growth regulated oncogene-α (CXCL1)	3505	GCST004457
IP10	Interferon gamma-induced protein 10 (CXCL10)	3685	GCST004440
MCP1	Monocyte chemotactic protein-1 (CCL2)	8293	GCST004438
MCP3	Monocyte specific chemokine 3 (CCL7)	843	GCST004437
MIG	Monokine induced by interferon-gamma (CXCL9)	3685	GCST004435
MIP1a	Macrophage inflammatory protein-1α (CCL3)	3522	GCST004434
MIP1b	Macrophage inflammatory protein-1β (CCL4)	8243	GCST004433
RANTES	Regulated on Activation, Normal T Cell Expressed and Secreted (CCL5)	3421	GCST004431
SDF1a	Stromal cell-derived factor-1 alpha (CXCL12)	5998	GCST004427
*Growth factors*			
bNGF	Beta nerve growth factor	3531	GCST004421
FGFbasic	Basic fibroblast growth factor	7565	GCST004459
GCSF	Granulocyte colony-stimulating factor	7904	GCST004458
HGF	Hepatocyte growth factor	8292	GCST004449
MCSF	Macrophage colony-stimulating factor	840	GCST004436
PDGFbb	Platelet-derived growth factor BB	8293	GCST004432
SCF	Stem cell factor	8290	GCST004429
SCGFb	Stem cell growth factor beta	3682	GCST004428
VEGF	Vascular endothelial growth factor	7118	GCST004422
*Interleukins*			
IL10	Interleukin-10	7681	GCST004444
IL12p70	Interleukin-12p70	8270	GCST004439
IL13	Interleukin-13	3557	GCST004443
IL16	Interleukin-16	3483	GCST004430
IL17	Interleukin-17	7760	GCST004442
IL18	Interleukin-18	3636	GCST004441
IL1b	Interleukin-1-beta	3309	GCST004448
IL1ra	Interleukin-1 receptor antagonist	3638	GCST004447
IL2	Interleukin-2	3475	GCST004455
IL2ra	Interleukin-2 receptor, alpha subunit	3677	GCST004454
IL4	Interleukin-4	8124	GCST004453
IL5	Interleukin-5	3364	GCST004452
IL6	Interleukin-6	8189	GCST004446
IL7	Interleukin-7	3409	GCST004451
IL8	Interleukin-8 (CXCL8)	3526	GCST004445
IL9	Interleukin-9	3634	GCST004450
*Others*			
IFNg	Interferon-gamma	7701	GCST004456
MIF	Macrophage migration inhibitory factor (glycosylation-inhibiting factor)	3494	GCST004423
TNFa	Tumor necrosis factor-alpha	3454	GCST004426
TNFb	Tumor necrosis factor-beta	1559	GCST004425
TRAIL	TNF-related apoptosis inducing ligand	8186	GCST004424

The meta-analysis focused on epilepsy and included data on focal epilepsy from 9671 cases and 29,677 controls of European descent and generalized epilepsy from 3769 cases and 29,677 controls of European descent.^[18]^ Patients diagnosed with focal and generalized epilepsy were classified according to the standard ILAE criteria. GWAS summary statistics were derived from the ILAE Consortium on Complex Epilepsies 2018. The GWAS exposure data were publicly available online through the IEU Open GWAS project.^[19]^ Link: https://gwas.mrcieu.ac.uk/. There is no crossover between the exposure and outcome groups.

### 2.3. Instrumental variable selection

Initially, we selected single nucleotide polymorphisms (SNPs) strongly associated with epilepsy and inflammatory cytokines using *P* < 5 × 10^−8^ as the genome-wide significance threshold. As very few SNPs were identified for some cytokines and epilepsy as exposures, a looser cutoff (*P* < 5 × 10^−6^) was chosen. Subsequently, we clustered these SNPs (kb = 10,000, r2 = 0.001) to avoid linkage disequilibrium. Then, palindromic SNPs were excluded because their linkage in the same direction for exposures and outcomes in the inflammatory cytokine GWASs could not be guaranteed. Additionally, to avoid any effects of weak instruments, the F-statistic was utilized to evaluate the instrument’s strength, and the R2 measured the variance in exposure for each SNP.^[20,21]^

### 2.4. Statistical analysis

To explore the causal relationship between inflammatory cytokines and epilepsy, we carried out a MR analysis. The Wald ratio (the ratio of genetic outcome associations to genetic exposure associations) was used to determine the correlation between the identified variable and epilepsy for traits that contained only one independent variable.^[22]^ Five commonly used MR methods were employed for functions that included several instrumental variables (IVs): the inverse variance weighted (IVW) test,^[23]^ the weighted mode,^[24]^ the MR-Egger,^[25]^ the weighted median,^[26]^ and the simple mode.^[27]^ For this reason, findings from studies that used more than one instrumental variable are based primarily on IVW, while the other 4 methods are complementary.^[26]^

In addition, several sensitivity analyses were conducted to evaluate the strength of the findings. The two-sample MR package and Cochran Q statistic were used to test for instrument heterogeneity. A Q greater than the number of instruments minus one indicates heterogeneity, and invalid instruments or significant Q statistics with a *P*-value < 0.05 may indicate heterogeneity.^[28,29]^ The MR-PRESSO technique provided that more than 50% of the instruments were validated and could locate an outlying genetic variant with horizontal pleiotropy.^[30]^ Moreover, a leave-one-out analysis was conducted to investigate whether a singular SNP was responsible for the causative effect. To assess the bias due to weak instruments, we also calculated the F-statistic.^[31]^ F-values below 10 are an indication of a weak instrument and are excluded. Two-sample MR^[19]^ and MR-PRESSO^[30]^ were utilized for all statistical analyses. In cases with a lack of pleiotropy and heterogeneity, a positive outcome for the IVW result should be considered as long as it is statistically significant at *P* < .05. This holds even if the other methods used do not demonstrate statistical significance, provided that the direction of the beta values aligns with that of the IVW result.^[32]^ Due to the number of inflammatory cytokines examined, we applied a Bonferroni correction (*P* < .0012, Bonferroni correction with 41 tests). Results satisfying *P* < .05 but not *P* < .0012 indicate suggestive associations.

### 2.5. Reverse MR analysis

To investigate the potential causal effect of epilepsy on inflammatory cytokines, a reverse MR analysis was performed using SNPs associated with focal epilepsy and generalized epilepsy as covariates, with focal epilepsy and generalized epilepsy defined as exposures and inflammatory cytokine levels as outcomes.

## 3. Results

### 3.1. Influence of 41 inflammatory cytokines on epilepsy

For 3 of the 41 inflammatory cytokines, a genome-wide significance cutoff of 5 × 10^−8^ resulted in 3 or more viable genetic variants. Subsequently, to guarantee sufficient SNPs for subsequent MR analysis, a higher cutoff of *P* < 5 × 10^−6^ was applied to the remaining cytokines. All F-statistic values exceeded 10, suggesting insignificant, weak instrumental bias^[33]^ (Table S1, Supplemental Digital Content, http://links.lww.com/MD/M633).

**Figure 2.**
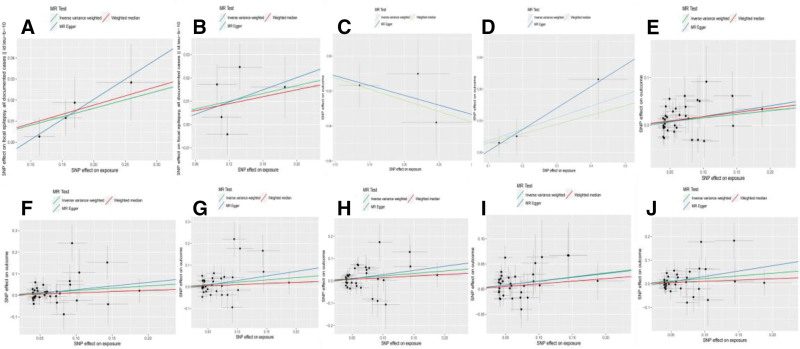
(A) Scatter plots for IL-5 on focal epilepsy. (B) Scatter plots for IL-ra on focal epilepsy. (C) Scatter plots for RANTES on generalized epilepsy. (D) Scatter plots for MCSF on generalized epilepsy. (E) Scatter plot for the outcome of IL-10. (F) Scatter plot for the outcome of IL-1b. (G) Scatter plot for the outcome of IL-1ra. (H) Scatter plot for the outcome of IL-7. (I) Scatter plot for the outcome of IFN-γ. (J) Scatter plot for the outcome of TNF-α.

All cytokines were examined using the IVW approach as the principal analytical technique. IVWs indicate that genetically increased IL-5 levels may increase the likelihood of developing focal epilepsy (OR: 1.084, 95% CI: 1.015–1.157, *P* = .015). It was determined using the weighted median approach, with the following results: OR = 1.091, 95%CI: 1.005–1.182, and *P* = .027. The analysis by MR Egger did not show a significant statistical correlation but did indicate a similar tendency to change. A causality between IL-1Ra and focal epilepsy has also been established. (IVW: OR = 1.087,95%CI: 1.003–1.179, *P* = .043) (Table 2). Figure 2 illustrates the scatter plots of MR analyses of IL-1Ra and IL-5 in focal epilepsy. The associations of IL-1Ra and IL-5 showed no evidence of heterogeneity as measured by Cochrane Q and I2 (I2: 0%; *P* = .824; I2: 0%; *P* = .208) and no outlier SNPs were discovered by MR-PRESSO. There was also no indication of directional pleiotropic effects (*P* = .482; *P* = .861) using the MR Egger intercept. See Table S1, Supplemental Digital Content, http://links.lww.com/MD/M633 for details of the SNPs. Figure S1, Supplemental Digital Content, http://links.lww.com/MD/M784 shows leave-one-out sensitivity analyses, forest plots, and funnel plots for IL-5 and IL-1Ra in focal epilepsy with MR.

**Table 2:** The result of *P* value, heterogeneity and horizontal pleiotropy of the cytokines and focal epilepsy in the forward MR analysis.

Exposures	nSNPs	b/se	OR (95%Cl)	pval	Het. pval	Ple. pval	MR-PRESSO
*Chemokines*							
CTACK	6	–0.027/0.021	0.974 (0.935,1.015)	0.205	0.725	0.878	0.794
Eotaxin	6	–0.030/0.057	0.970 (0.867,1.086)	0.598	0.106	0.852	0.158
GROa	3	–0.017/0.022	0.983 (0.941,1.027)	0.439	0.369	0.669	NA
IP10	23	0.013/0.017	0.987 (0.955,1.020)	0.427	0.373	0.132	0.373
MCP1	6	0.041/0.043	1.042 (0.958,1.132)	0.340	0.316	0.427	0.330
MCP3	30	–0.003/0.010	0.997 (0.978,1.016)	0.726	0.046	0.535	0.048
MIG	5	0.012/0.029	1.012 (0.955,1.071)	0.694	0.583	0.551	0.660
MIP1a	3	–0.083/0.042	1.086 (1.000,1.180)	0.050	0.888	0.712	NA
MIP1b	4	–0.028/0.040	0.972 (0.898,1.052)	0.483	0.885	0.553	0.970
RANTES	3	0.009/0.074	1.009 (0.873,1.166)	0.903	0.019	0.572	NA
SDF1a	3	0.053/0.048	1.054 (0.960,1.158)	0.269	0.890	0.742	NA
*Growth factors*							
bNGF	22	0.009/0.020	1.009 (0.969,1.049)	0.674	0.031	0.485	0.033
FGFbasic	30	–0.036/0.026	0.965 (0.917,1.015)	0.168	0.008	0.774	0.007
GCSF	26	0.023/0.031	1.024 (0.963,1.088)	0.455	0.017	0.752	0.019
HGF	4	0.076/0.088	1.079 (0.908,1.281)	0.388	0.065	0.143	0.191
MCSF	3	–0.032/0.034	0.969 (0.906,1.036)	0.354	0.932	0.791	NA
PDGFbb	3	0.052/0.042	1.053 (0.971,1.143)	0.213	0.659	0.733	NA
SCF	5	0.031/0.037	1.032 (0.959,1.110)	0.404	0.327	0.807	0.427
SCGFb	5	–0.005/0.048	0.995 (0.906,1.093)	0.918	0.022	0.848	0.110
VEGF	4	0.022/0.016	1.023 (0.991,1.055)	0.163	0.801	0.869	0.914
*Interleukins*							
IL10	11	0.030/0.022	1.030 (0.986,1.076)	0.186	0.947	0.871	0.956
IL12p70	5	0.021/0.021	1.021 (0.980,1.064)	0.328	0.875	0.392	0.653
IL13	5	0.019/0.026	1.019 (0.969,1.071)	0.467	0.104	0.959	0.326
IL16	24	0.023/0.013	1.023 (0.998,1.048)	0.071	0.516	0.073	0.558
IL17	6	0.050/0.038	1.051 (0.975,1.133)	0.193	0.741	0.556	0.792
IL18	6	–0.006/0.026	0.994 (0.945,1.045)	0.809	0.216	0.706	0.240
IL1b	25	–0.001/0.020	0.999 (0.961,1.038)	0.947	0.020	0.291	0.021
IL1ra	5	0.083/0.041	1.087 (1.003,1.179)	**0.043**	0.208	0.861	0.269
IL2	5	–0.022/0.035	0.978 (0.914,1.047)	0.527	0.502	0.220	0.538
IL2ra	4	–0.001/0.041	0.999 (0.923,1.082)	0.983	0.941	0.594	0.943
IL4	8	–0.003/0.054	0.997 (0.896,1.109)	0.954	0.015	0.041	0.030
IL5	4	0.080/0.033	1.084 (1.015,1.157)	**0.015**	0.824	0.482	0.861
IL6	3	0.082/0.057	1.085 (0.970,1.213)	0.152	0.784	0.713	NA
IL7	5	0.023/0.027	1.023 (0.969,1.079)	0.412	0.171	0.492	0.332
IL8	4	0.003/0.025	1.003 (0.954,1.053)	0.920	0.793	0.871	0.842
IL9	3	0.031/0.039	1.032 (0.956,1.053)	0.422	0.919	0.781	NA
*Others*							
IFNg	3	0.038/0.065	1.038 (0.914,1.180)	0.564	0.493	0.447	NA
MIF	2	0.223/0.123	1.250 (0.981,1.592)	0.071	NA	NA	NA
TNFa	18	–0.001/0.018	1.001 (0.967,1.037)	0.935	0.927	0.655	0.926
TNFb	20	–0.007/0.013	0.993 (0.968,1.018)	0.571	0.300	0.425	0.301
TRAIL	6	–0.006/0.032	1.006 (0.945,1.071)	0.851	0.121	0.735	0.309

Bold values are statistically significant (*P* < 0.05).

For generalized epilepsy, IVWs indicate that genetically increased regulated upon activation normal T cell expressed and secreted factor (RANTES) levels may increase the likelihood of developing generalized epilepsy (OR: 1.116, 95% CI: 1.004–1.241, *P* = .042). In addition, IVWs indicate that genetically increased MCSF levels may decrease the likelihood of developing generalized epilepsy (OR: 0. 894, 95% CI: 0.811–0.985, *P* = .024). It was determined using the weighted median approach, with the following results: OR = 0.881, 95%CI: 0.779–0.996, and *P* = .043. The analysis by MR Egger did not show a significant statistical correlation but did indicate a similar tendency to change (Table S2, Supplemental Digital Content, http://links.lww.com/MD/M634). The associations of RANTES and MCSF showed no evidence of heterogeneity, and MR-PRESSO discovered no outlier SNPs. There was also no indication of directional pleiotropic effects. Figure 2 illustrates the scatter plots of RANTES and MCSF MR analyses in generalized epilepsy. Figure S1, Supplemental Digital Content, http://links.lww.com/MD/M784 shows leave-one-out sensitivity analyses, forest plots, and funnel plots for RANTES and MCSF in generalized epilepsy with MR.

### 3.2. Influence of epilepsy on 41 inflammatory cytokines

**Figure 3.**
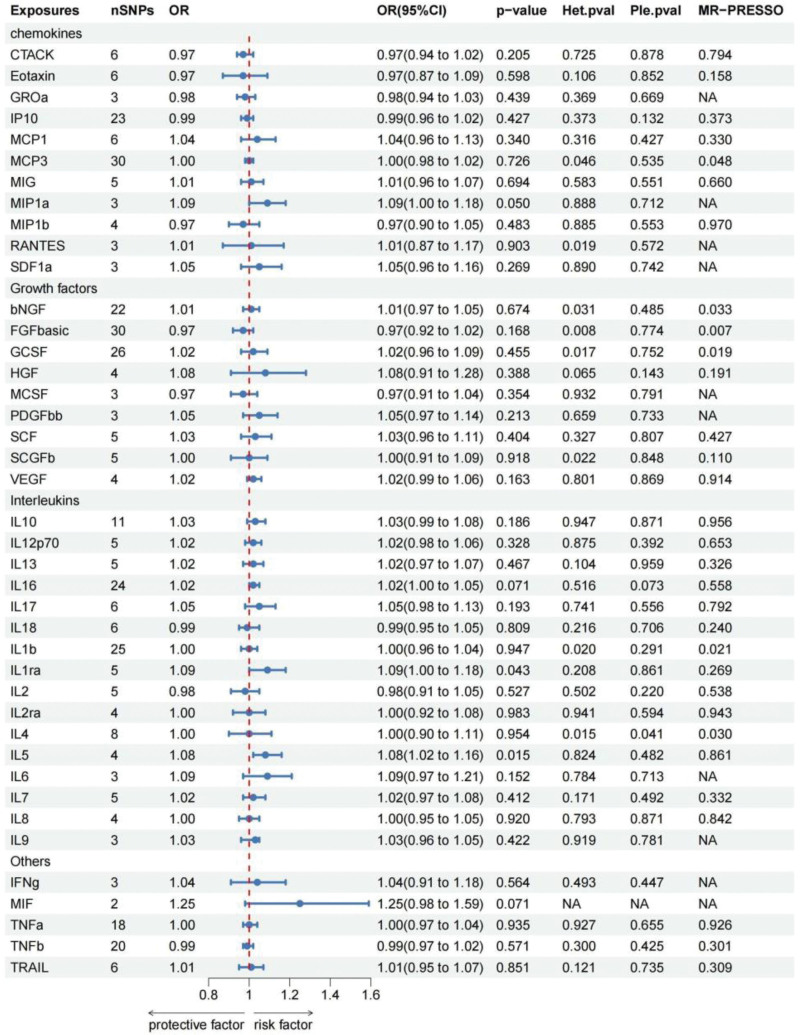
Forest plots of the causal relationship between 41 inflammatory factors and focal epilepsy in the result of IVW in the forward MR analysis.

**Figure 4.**
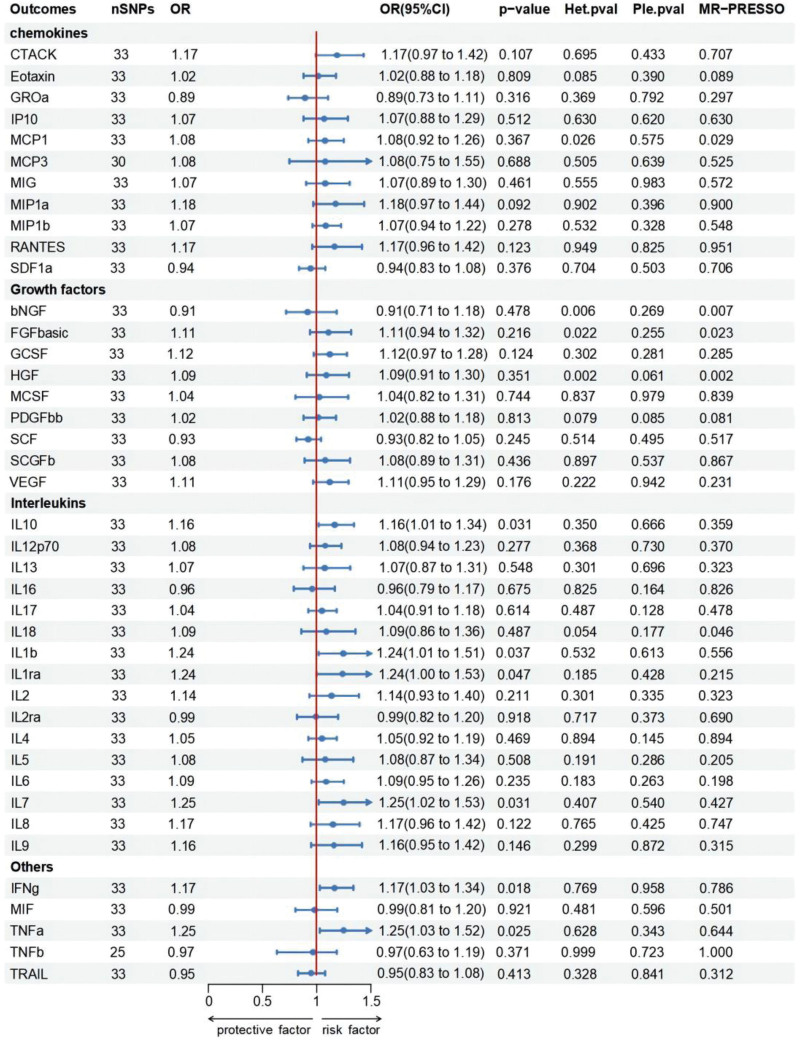
Forest plots of the causal relationship between focal epilepsy and 41 inflammatory factors in the result of IVW in the reverse MR analysis.

To obtain more relevant SNPs, we used a genome-wide significance threshold of *P* < 5 × 10^−6^ to select SNPs highly correlated with focal epilepsy. Similarly, genetically predicted focal epilepsy was associated with increased levels of several cytokines, including IL-10, IL-1β, IL-1Ra, IL-7, interferon-γ (IFN-γ), and TNF-α (IVW: OR = 1.164, 95% CI = 1.014–1.337, *P* = .031; IVW: OR = 1.239, 95% CI = 1.013–1.514, *P* = .037; IVW: OR = 1.239, 95% CI = 1.003–1.530, *P* = .047; IVW: OR = 1.248, 95% CI = 1.021–1.527, *P* = .031; IVW: OR = 1.174, 95% CI = 1.028–1.341, *P* = .018; IVW: OR = 1.251, 95% CI = 1.028–1.523, *P* = .025). The validity of the IVW method was also confirmed by sensitivity tests using MR-PRESSO, Cochran Q, and the MR-Egger intercept, which showed no statistically meaningful findings. Furthermore, the results of the MR and sensitivity analyses concerning the prediction of cytokines and focal epilepsy are presented in Table 3. The scatter plots illustrating the findings above are shown in Figure 2, while Table S1, Supplemental Digital Content, http://links.lww.com/MD/M633 provides information on the SNPs. Figure S1, Supplemental Digital Content, http://links.lww.com/MD/M784 shows leave-one-out sensitivity analyses, forest plots, and funnel plots for focal epilepsy in IL-10, IL-1Ra, IL-7, TNF-α, IFN-γ, and IL-1β with MR. Figures 3 and 4 present the findings of the MR and sensitivity analyses concerning focal epilepsy and cytokines. However, we have not found which inflammatory cytokines are altered in generalized epilepsy (Table S3, Supplemental Digital Content, http://links.lww.com/MD/M635).

**Table 3:** The result of *P* value, heterogeneity and horizontal pleiotropy of the focal epilepsy and cytokines in the reverse MR analysis.

Outcomes	nSNPs	b/se	OR (95%Cl)	pval	Het. pval	Ple. pval	MR-PRESSO
*Chemokines*							
CTACK	33	0.157/0.098	1.171 (0.967,1.418)	0.107	0.695	0.433	0.707
Eotaxin	33	0.019/0.077	1.019 (0.876,1.184)	0.809	0.085	0.390	0.089
GROa	33	–0.106/0.105	0.890 (0.732,1.106)	0.316	0.369	0.792	0.297
IP10	33	0.064/0.097	1.066 (0.881,1.289)	0.512	0.630	0.620	0.630
MCP1	33	0.073/0.081	1.076 (0.918,1.262)	0.367	0.026	0.575	0.029
MCP3	30	0.075/0.186	1.077 (0.749,1.550)	0.688	0.505	0.639	0.525
MIG	33	0.071/0.097	1.074 (0.888,1.299)	0.461	0.555	0.983	0.572
MIP1a	33	0.167/0.099	1.182 (0.973,1.436)	0.092	0.902	0.396	0.900
MIP1b	33	0.071/0.065	1.074 (0.944,1.220)	0.278	0.532	0.328	0.548
RANTES	33	0.156/0.101	1.169 (0.959,1.424)	0.123	0.949	0.825	0.951
SDF1a	33	–0.060/0.067	0.942 (0.825,1.075)	0.376	0.704	0.503	0.706
*Growth factors*							
bNGF	33	–0.093/0.131	0.911 (0.705,1.178)	0.478	0.006	0.269	0.007
FGFbasic	33	0.106/0.085	1.111 (0.940,1.314)	0.216	0.022	0.255	0.023
GCSF	33	0.018/0.070	1.114 (0.971,1.279)	0.124	0.302	0.281	0.285
HGF	33	0.084/0.090	1.087 (0.912,1.297)	0.351	0.002	0.061	0.002
MCSF	33	0.039/0.119	1.040 (0.823,1.313)	0.744	0.837	0.979	0.839
PDGFbb	33	0.018/0.077	1.018 (0.876,1.183)	0.813	0.079	0.085	0.081
SCF	33	–0.076/0.065	0.927 (0.816,1.053)	0.245	0.514	0.495	0.517
SCGFb	33	0.076/0.097	1.079 (0.892,1.305)	0.436	0.897	0.537	0.867
VEGF	33	0.104/0.077	1.109 (0.954,1.289)	0.176	0.222	0.942	0.231
*Interleukins*							
IL10	33	0.152/0.070	1.164(1.014,1.337)	**0.031**	0.350	0.666	0.359
IL12p70	33	0.073/0.067	1.076 (0.943,1.228)	0.277	0.368	0.730	0.370
IL13	33	0.063/0.104	1.065 (0.868,1.306)	0.548	0.301	0.696	0.323
IL16	33	–0.042/0.100	0.959 (0.789,1.166	0.675	0.825	0.164	0.826
IL17	33	0.034/0.068	1.035 (0.906,1.181)	0.614	0.487	0.128	0.478
IL18	33	0.081/0.117	1.085 (0.863,1.364)	0.487	0.054	0.177	0.046
IL1b	33	0.214/0.102	1.239 (1.013,1.514)	**0.037**	0.532	0.613	0.556
IL1ra	33	0.214/0.108	1.239 (1.003,1.530)	**0.047**	0.185	0.428	0.215
IL2	33	0.132/0.105	0.141 (0.928,1.403)	0.211	0.301	0.335	0.323
IL2ra	33	–0.010/0.097	0.990 (0.818,1.198)	0.918	0.717	0.373	0.690
IL4	33	0.048/0.066	1.049 (0.922,1.194)	0.469	0.894	0.145	0.894
IL5	33	0.074/0.112	1.077 (0.865,1.340)	0.508	0.191	0.286	0.205
IL6	33	0.086/0.073	1.090 (0.945,1.257)	0.235	0.183	0.263	0.198
IL7	33	0.222/0.103	1.248 (1.021,1.527)	**0.031**	0.407	0.540	0.427
IL8	33	0.153/0.099	1.166 (0.960,1.416)	0.122	0.765	0.425	0.747
IL9	33	0.150/0.103	1.162 (0.949,1.422)	0.146	0.299	0.872	0.315
*Others*							
IFNγ	33	0.161/0.068	1.174 (1.028,1.341)	**0.018**	0.769	0.958	0.786
MIF	33	–0.010/0.100	0.990 (0.814,1.204)	0.921	0.481	0.596	0.501
TNFa	33	0.224/0.100	1.251 (1.028,1.523)	**0.025**	0.628	0.343	0.644
TNFb	25	–0.145/0.162	0.865 (0.629,1.189)	0.371	0.999	0.723	1.000
TRAIL	33	–0.056/0.069	0.945 (0.827,1.081)	0.413	0.328	0.841	0.312

Bold values are statistically significant (*P* < 0.05).

## 4. Discussion

Epilepsy is a prevalent neurological disorder impacting about 70 million individuals globally.^[34]^ The challenge of assessing susceptibility and risk, inadequate and delayed access to care, and poor patient compliance are among the current difficulties in managing epilepsy. An association between epileptic seizures and inflammatory responses in specific brain regions has been suggested by recent clinical and experimental studies.^[35,36]^

We discovered a bidirectional causality between cytokines and epilepsy by investigating 41 cytokines in the most comprehensive GWAS datasets. Specifically, focal epilepsy onset may induce increases in cytokines such as IL-7, IL-1Ra, IL-10, TNF-α, IFN-γ, and IL-1β, whereas inconsistent cytokine levels, including IL-5 and IL-1ra, may influence variations in focal epilepsy risk. In addition, RANTES and MCSF may be associated with the development of generalized epilepsy. This study is the most extensive MR investigation of the causative link between epilepsy and inflammatory regulators.

### 4.1. Causal link between pro-inflammatory factors and epilepsy

**Figure 5.**
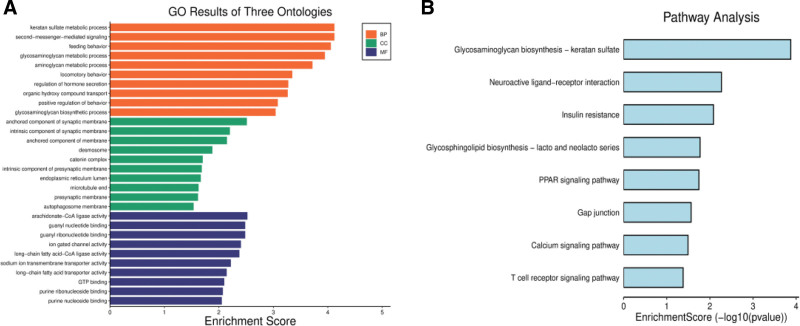
The GO and KEGG analyses of IL5 on focal epilepsy. Gene ontology (GO); Kyoto Encyclopedia of Genes and Genomes (KEGG); molecular function (MF); cellular component (CC); biological process (BP).

IL-5 is an important molecule in various processes essential for maintaining good health and directly or indirectly linked to disease development. Specific pharmacological agents can affect the production of IL-5 in vivo. Our study has shown a positive association between IL-5 and the development of focal epilepsy. Previous observational studies have shown a significant increase in IL-5 in patients with epilepsy (especially in those with drug-resistant epilepsy). It is consistent with our findings.^[9]^ However, the underlying mechanisms are poorly known. To investigate the underlying mechanisms, we performed bioinformatic analyses to identify critical genes that might be associated with epilepsy through IL-5. We examined the human brain microarray dataset GSE31718 from the GEO using the bioinformatics method to identify differentially expressed genes between epilepsy patients and normal subjects. These differentially expressed genes were functionally annotated using a multi-step bioinformatics approach, including gene ontology and the Kyoto Encyclopedia of Genes and Genomes analysis. Kyoto Encyclopedia of Genes and Genomes and gene ontology analyses consistently indicated that IL5 is primarily involved in metabolic processes, behavioral regulation, and synthesis of synaptic membrane components in focal epilepsy (Fig. 5). Further confirmation of the exact mechanisms of action by in vitro experiments is necessary.

RANTES is a crucial chemokine. Previous observational studies have shown that it is strongly associated with the development of epilepsy.^[37]^ The results of our MR study are in line with the findings of previous studies. Activation of the CCL5/CCR5 pathway may be involved in RANTES’ involvement in epileptogenesis.^[38]^ In addition, RANTES may be associated with microglial activation and proliferation,^[39]^ increased excitotoxins, and disrupted blood-brain barrier.^[40]^ By reducing CCR5 in circulating cells, we can protect against excitotoxin-induced seizures, blood-brain barrier leakage, CNS damage, and inflammation and promote neurogenic repair.

IL-1β is a typical pro-inflammatory cytokine. Based on our research, many observational studies show that people with focal epilepsy have elevated levels of IL-1β. This cytokine could affect GABAergic neurotransmission in brain tissue, thereby affecting excitability during seizures.^[41]^ In medial temporal lobe epilepsy, IL-1β is implicated in focal epileptogenesis by activating the PI3K/Akt/mTOR signaling pathway.^[42]^ As a result, IL-1β could serve as a significant biomarker for focal epilepsy. Additionally, IL-1β could promptly reduce the threshold of focal ictal events and enhance the likelihood that targeting these signaling pathways could prove an efficient therapeutic approach in preventing focal epilepsy. TNF-α is a pleiotropic cytokine regulating cell survival, inflammation, and death. Previous studies have suggested a significant increase in TNF-α in focal epilepsy, and our MR study supports this. TNF-α promotes focal epileptogenesis development by increasing hyperexcitability through autocrine mechanisms such as astrocyte glutamate release, modulation of neuronal transmitter release, and purinergic signaling.^[43]^ The pro-inflammatory cytokine TNF-α may be associated with iron regulation and metabolism in the progression of epilepsy and may act as an acute seizure biomarker.^[44]^

### 4.2. Causal link between anti-inflammatory factors and epilepsy

IL-1Ra acts as an endogenous antagonist of IL-1R. The MR analysis performed in this study shows that IL-1Ra is present both upstream and downstream of focal epilepsy. Focal epilepsy leads to significant increases in IL-1Ra, and higher levels of IL-1Ra also promote focal epilepsy. Our research supports previous observational studies and reinforces their conclusions. Several studies have shown elevated levels of IL-1Ra in focal epilepsy.^[45,46]^ Such levels of IL-1Ra may help the nervous system protect itself against excitotoxic neuronal damage.^[47]^ Research has found that IL-1RA enhances epileptic refractoriness in a mouse model of hippocampal ignition, and this evidence points to the increased presence of IL-Ra as a contributing factor to focal epileptogenesis, as the hippocampus tends to be a common site of epileptogenesis in the temporal lobes.^[48]^ The exact mechanism for this has yet to be discovered. However, research suggests a possible association between the IL-1Ra gene and temporal lobe epilepsy due to genetic polymorphisms.^[49]^ These findings provide a credible explanation for the observed results and identify IL-Ra as an essential biomarker for focal epilepsy.

In our MR study, patients diagnosed with focal epilepsy exhibited heightened levels of IL-10, a vital anti-inflammatory cytokine. IL-10 proved essential in reinforcing GABAergic currents while inhibiting IL-10 signaling, thereby contributing to the suppression of focal epilepsy. Notably, IL-1β decreases GABA currents’ amplitude and impedes the efficacy of IL-10.^[50]^ Focal epilepsy is believed to elevate IL-10 as a possible safeguarding mechanism in the brain that mitigates inflammatory harm. It is noted that IL-10 secretion is subject to seizure frequency and depletes with the progression of the disease. Consequently, evaluating plasma IL-10 levels can serve as a prospective biomarker/marker to detect the onset of progressive epilepsy.^[51]^

### 4.3. Causal link between regulatory cytokines and epilepsy

MCSF is a critical growth factor that controls macrophages and monocytes’ renewal, survival, differentiation, and proliferation. In our MR study, MCSF was negatively associated with generalized epilepsy. M-CSF can modulate immunity and protect neurons by acting on the receptors of adult microglia to regulate the expression of receptors and transcription factors.^[52]^ Therefore, by targeting MCSF-related targets, we may be able to treat epilepsy in the future. IL-7 is a pluripotent cytokine that is essential for the maintenance of immune system homeostasis. It plays a role in T cells’ development, differentiation, proliferation, and the maturation of B cells by activating IL-7R. Consistent with our study, previous research has shown that IL-7 is elevated in those with focal epilepsy.^[53]^ It has been shown that partial focal epilepsy can be treated with lamotrigine. This reduces the in vivo expression levels of IL-7.^[54]^ This means that IL-7 is an essential biomarker in focal epilepsy, which indicates that inflammation may have a vital function for focal epilepsy. Reducing IL-7 using drugs may help treat focal epilepsy.

IFN-γ is a vital immunomodulatory cytokine generated by activated T and NK cells, crucial in supporting the host’s immune defense. Numerous observational studies have reported extensive IFN-γ elevation among epilepsy patients, including our study. The mechanism behind epilepsy development and IFN-γ’s ability to augment the permeability of the small bowel epithelial barriers and blood-brain is associated positively.^[55]^ In addition, research has indicated that it may affect the proportion of microglia.^[56]^ Studies have found that by targeting miR-29a-HMGB1, which regulates neuronal apoptosis and neuroinflammation and thereby IFN-γ release, temporal lobe epilepsy can be controlled.^[57]^ IFN-γ can indicate that epilepsy is not healing correctly. Therefore, it is a significant biomarker for focal epilepsy. Focal epilepsies can be treated by targeting miR-29a-HMGB1-IFN-γ.

### 4.4. Limitations

Although the study is significant, it has some limitations. All those involved in the GWAS were European-born. Whether our findings can be applied to other regions and populations remains to be seen. Furthermore, MR was found to have numerous biases, such as differential survival bias and selection bias, with population stratification bias being the most frequent. In addition, performing disaggregated analyses based on these markers is hampered by the lack of categorized information on the characteristics of patients with epilepsy, including age at onset, gender, and disease severity (e.g., frequency and duration of seizures). However, more comprehensive data are needed to achieve more evidence to improve the present knowledge of seizure subtypes in epilepsy.

## 5. Conclusion

In conclusion, this MR study demonstrates that interleukin-5 and interleukin-1 receptor antagonists are potentially correlated factors with focal epilepsy etiology, while interleukin-10, interleukin-1β, interleukin-1Ra, interleukin-7, tumor necrosis factor-α, and interferon-γ possibly contribute to focal epilepsy development downstream. In addition, MCSF and RANTES are potentially correlated factors with generalized epilepsy etiology. Therefore, targeting bioactive factors and changing body cytokine levels can significantly reduce the recurrence and refractory nature of epilepsy.

## Acknowledgments

We thank all the genetics consortiums for making the GWAS summary data publicly available.

## Author contributions

**Conceptualization:** Wencai Wang, Luyao Ma, Menghao Liu, Yongqiang Zhao, Wei Ye, Xianfeng Li.

**Data curation:** Wencai Wang.

**Formal analysis:** Wencai Wang.

**Funding acquisition:** Xianfeng Li.

**Investigation:** Wencai Wang.

**Methodology:** Wencai Wang.

**Project administration:** Wencai Wang.

**Resources:** Wencai Wang.

**Software:** Wencai Wang.

**Supervision:** Wencai Wang.

**Validation:** Wencai Wang.

**Visualization:** Wencai Wang.

**Writing – original draft:** Wencai Wang.

**Writing – review & editing:** Wencai Wang, Luyao Ma, Xianfeng Li.

## Supplementary Material








