# Supplementary material for: Unraveling the links between circulating bioactive factors and epilepsy: A bidirectional Mendelian randomization study
Source: Medicine (Baltimore). 2024 May 31;103(22):e38256. doi: 10.1097/MD.0000000000038256 (PMC11142776; doi:10.1097/MD.0000000000038256)
Supplement: Supplementary file 2 [file medi-103-e38256-s002.docx]

Supplementary Figure: The funnel plots, forest plots and leave-one-out plot for exposures on outcomes

A


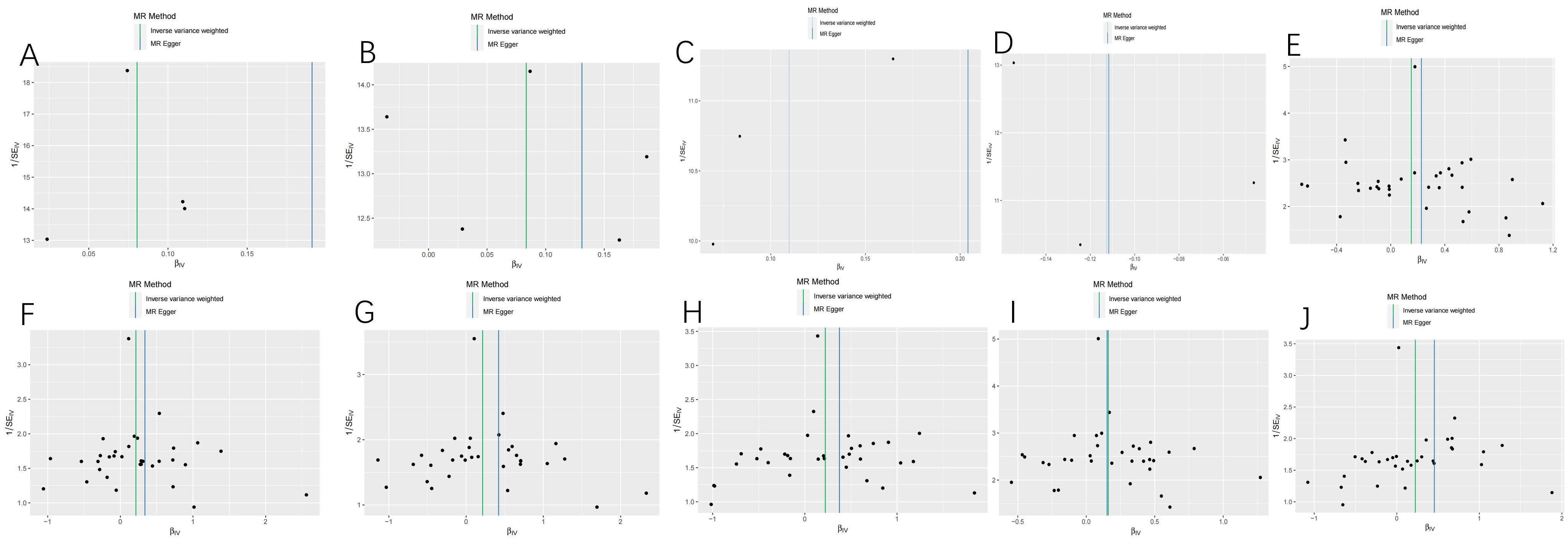


(A) funnel plot for IL-5 on focal epilepsy (B) funnel plot for IL-1Ra on focal epilepsy (C) funnel plot for RANTES on generalized epilepsy (D) funnel plot for MCSF on generalized epilepsy (E) funnel plot for the outcome of IL-10. (F) funnel plot for the outcome of IL-1b. (G) funnel plot for the outcome of IL-1ra. (H)funnel plot for the outcome of IL-7. (I) funnel plot for the outcome of IFN-γ. (J) funnel plot for the outcome of TNF-α

B


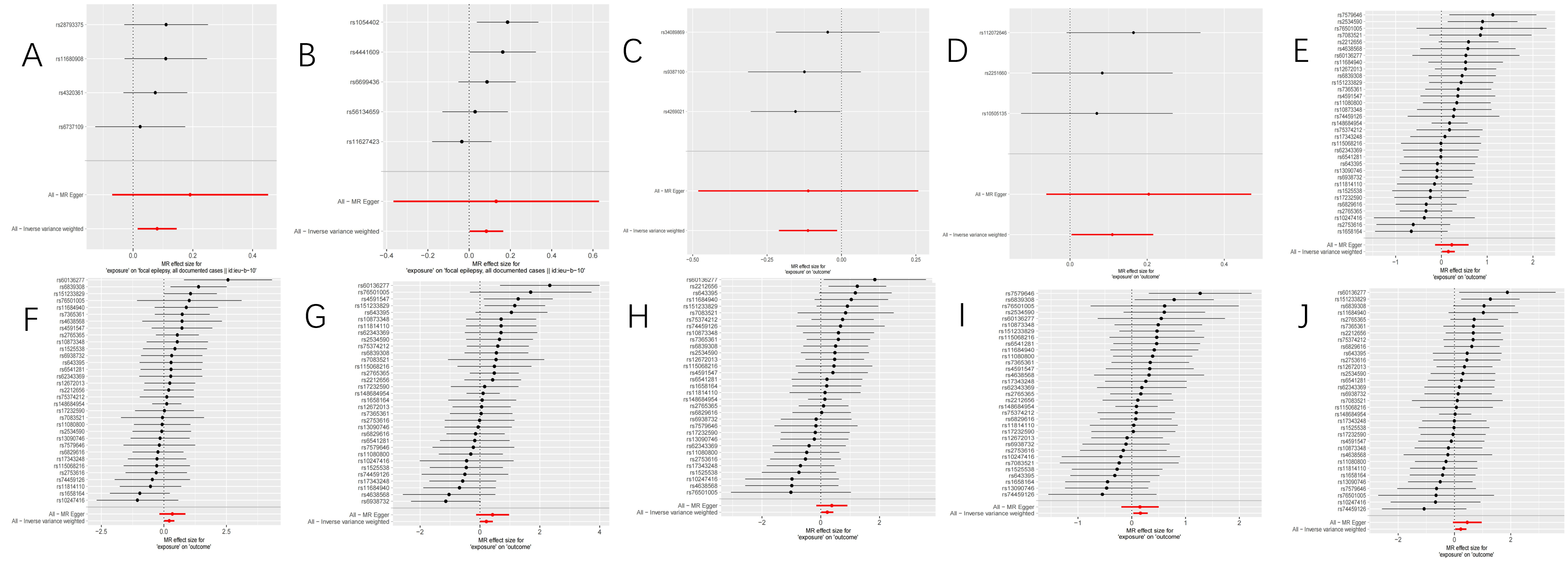


(A) forest plot for IL-5 on focal epilepsy (B) forest plot for IL-1Ra on focal epilepsy (C) forest plot for RANTES on generalized epilepsy (D) forest plot for MCSF on generalized epilepsy (E) forest plot for the outcome of IL-10. (F) forest plot for the outcome of IL-1b. (G) forest plot for the outcome of IL-1ra. (H) forest plot for the outcome of IL-7. (I) forest plot for the outcome of IFN-γ. (J) forest plot for the outcome of TNF-α


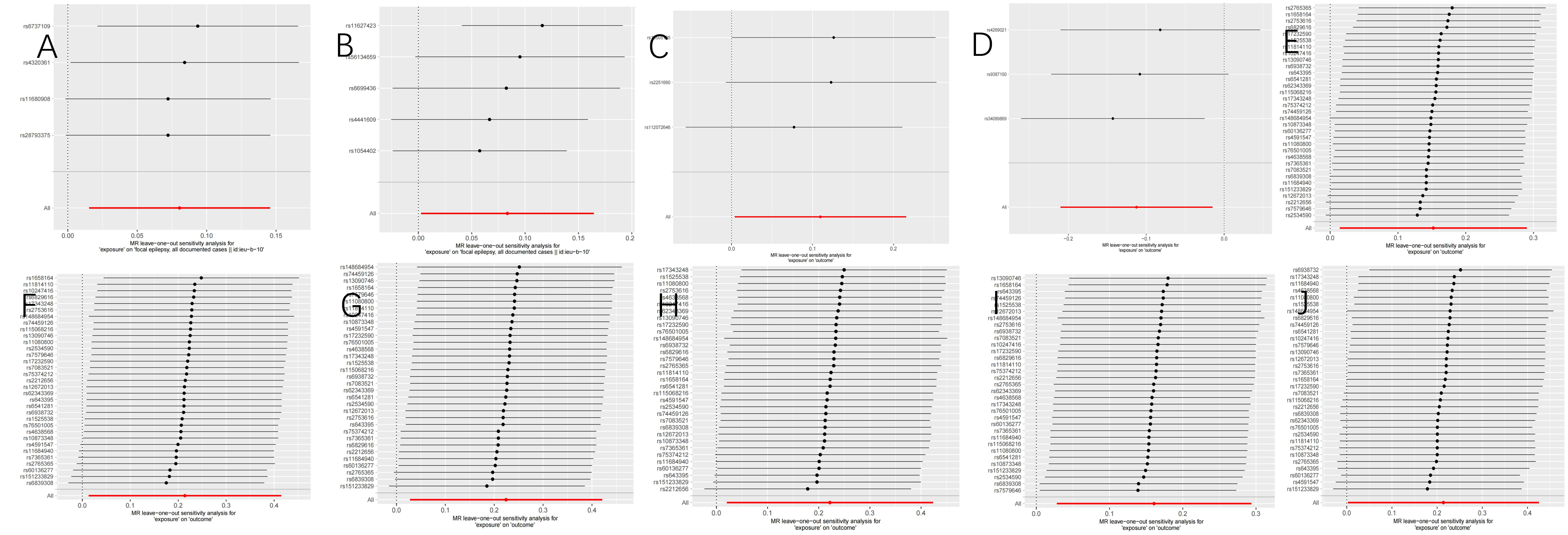


(A) leave-one-out plot for IL-5 on focal epilepsy (B) leave-one-out plot for IL-1Ra on focal epilepsy (C) leave-one-out plot for RANTES on generalized epilepsy (D) leave-one-out plot for MCSF on generalized epilepsy (E) leave-one-out plot for the outcome of IL-10. (F) leave-one-out plot for the outcome of IL-1b. (G) leave-one-out plot for the outcome of IL-1ra. (H) leave-one-out plot for the outcome of IL-7. (I) leave-one-out plot for the outcome of IFN-γ. (J) leave-one-out plot for the outcome of TNF-α
