# Supplementary material for: Unraveling the links between circulating bioactive factors and epilepsy: A bidirectional Mendelian randomization study
Source: Medicine (Baltimore). 2024 May 31;103(22):e38256. doi: 10.1097/MD.0000000000038256 (PMC11142776; doi:10.1097/MD.0000000000038256)
Supplement: Supplementary file 3 [file medi-103-e38256-s003.docx]

| **Exposures** | **nSNPs** | **b/se** | **OR(95%Cl)** | **pval** | **Het. pval** | **Ple.pval** | **MR-PRESSO** |
| --- | --- | --- | --- | --- | --- | --- | --- |
| **chemokines** | | | | | | | |
| CTACK | 6 | -0.006/0.035 | 0.994(0.928,1.066) | 0.875 | 0.229 | 0.539 | 0.339 |
| Eotaxin | 4 | 0.079/0.096 | 1.082(0.896,1.307) | 0.412 | 0.198 | 0.488 | 0.277 |
| GROa | 3 | 0.026/0.059 | 1.026(0.914,1.152) | 0.661 | 0.041 | 0.330 | NA |
| IP10 | 2 | -0.191/0.073 | 0.826(0.715,0.954) | 0.009 | 0.892 | NA | NA |
| MCP1 | 5 | 0.053/0.062 | 1.055(0.934,1.191) | 0.391 | 0.489 | 0.191 | 0.579 |
| MCP3 | 2 | 0.021/0.048 | 1.021(0.930,1.121) | 0.662 | 0.915 | NA | NA |
| MIG | 1 | 0.012/0.132 | 1.012(0.781,1.312) | 0.929 | NA | NA | NA |
| MIP1a | 3 | -0.071/0.080 | 0.932(0.796,1.090) | 0.379 | 0.669 | 0.448 | NA |
| MIP1b | 3 | -0.025/0.062 | 0.975(0.863,1.101) | 0.682 | 0.528 | 0.909 | NA |
| RANTES | 3 | 0.110/0.054 | 1.116(1.004,1.241) | **0.042** | 0.732 | 0.586 | NA |
| SDF1a | 3 | 0.002/0.080 | 1.002(0.857,1.173) | 0.975 | 0.249 | 0.935 | NA |
| **Growth factors** | | | | | | | |
| bNGF | 1 | -0.087/0.104 | 0.916(0.748,1.123) | 0.400 | NA | NA | NA |
| FGFbasic | 2 | -0.071/0.239 | 0.931(0.583,1.486) | 0.765 | 0.045 | NA | NA |
| GCSF | 2 | -0.059/0.239 | 0.943(0.590,1.507) | 0.805 | 0.035 | NA | NA |
| HGF | 3 | -0.060/0.132 | 0.941(0.727,1.220) | 0.648 | 0.099 | 0.302 | NA |
| MCSF | 3 | -0.113/0.050 | 0.894(0.811,0.985) | **0.024** | 0.647 | 0.997 | NA |
| PDGFbb | 8 | -0.062/0.048 | 0.939(0.854,1.033) | 0.197 | 0.473 | 0.416 | 0.535 |
| SCF | 5 | -0.031/0.072 | 0.969(0.842,1.115) | 0.662 | 0.225 | 0.798 | 0.262 |
| SCGFb | 5 | -0.010/0.086 | 0.990(0.837,1.171) | 0.906 | 0.002 | 0.712 | 0.048 |
| VEGF | 5 | 0.060/0.051 | 1.062(0.962,1.173) | 0.234 | 0.658 | 0.277 | 0.692 |
| **Interleukins** | | | | | | | |
| IL10 | 10 | 0.024/0.064 | 1.024(0.904,1.161) | 0.707 | 0.095 | 0.030 | 0.108 |
| IL12p70 | 5 | -0.005/0.031 | 0.995(0.937,1.057) | 0.872 | 0.485 | 0.935 | 0.711 |
| IL13 | 3 | -0.017/0.063 | 0.983(0.869,1.112) | 0.788 | 0.508 | 0.456 | NA |
| IL16 | 3 | -0.004/0.029 | 0.996(0.941,1.054) | 0.893 | 0.985 | 0.937 | NA |
| IL17 | 4 | -0.028/0.104 | 0.972(0.793,1.191) | 0.784 | 0.109 | 0.652 | 0.198 |
| IL18 | 6 | -0.008/0.033 | 0.992(0.930,1.059) | 0.816 | 0.338 | 0.666 | 0.420 |
| IL1b | 1 | -0.045/0.093 | 0.956(0.797,1.147) | 0.630 | NA | NA | NA |
| IL1ra | 4 | -0.003/0.084 | 0.997(0.846,1.176) | 0.976 | 0.067 | 0.923 | 0.133 |
| IL2 | 4 | 0.050/0.056 | 1.051(0.941,1.174) | 0.380 | 0.713 | 0.550 | 0.726 |
| IL2ra | 3 | 0.031/0.069 | 1.031(0.901,1.180) | 0.654 | 0.701 | 0.715 | NA |
| IL4 | 7 | -0.062/0.056 | 0.940(0.843,1.049) | 0.270 | 0.318 | 0.521 | 0.381 |
| IL5 | 4 | -0.018/0.049 | 0.982(0.893,1.081) | 0.717 | 0.917 | 0.576 | 0.934 |
| IL6 | 3 | -0.021/0.098 | 0.979(0.808,1.186) | 0.829 | 0.439 | 0.715 | NA |
| IL7 | 3 | -0.027/0.064 | 0.973(0.858,1.103) | 0.670 | 0.980 | 0.880 | NA |
| IL8 | 3 | 0.023/0.065 | 1.023(0.902,1.161) | 0.722 | 0.125 | 0.291 | NA |
| IL9 | 3 | 0.066/0.103 | 1.068(0.883,1.293) | 0.524 | 0.035 | 0.667 | NA |
| **Others** | | | | | | | |
| IFNg | 3 | -0.118/0.099 | 0.889(0.733,1.079) | 0.235 | 0.493 | 0.787 | NA |
| MIF | 2 | 0.112/0.143 | 1.130(0.854,1.494) | 0.393 | 0.068 | NA | NA |
| TNFa | 2 | -0.072/0.061 | 0.931(0.826,1.048) | 0.237 | NA | NA | NA |
| TNFb | 2 | 0.051/0.052 | 1.052(0.950,1.165) | 0.328 | NA | NA | NA |
| TRAIL | 6 | 0.002/0.038 | 1.002(0.929,1.080) | 0.968 | 0.325 | 0.640 | 0.488 |

Supplementary Table 2

The result of P value, heterogeneity and horizontal pleiotropy of the cytokines and generalized epilepsy in the forward MR analysis
