# Supplementary material for: Unraveling the links between circulating bioactive factors and epilepsy: A bidirectional Mendelian randomization study
Source: Medicine (Baltimore). 2024 May 31;103(22):e38256. doi: 10.1097/MD.0000000000038256 (PMC11142776; doi:10.1097/MD.0000000000038256)
Supplement: Supplementary file 4 [file medi-103-e38256-s004.docx]

| **Outcomes** | **nSNPs** | **b/se** | **OR(95%Cl)** | **pval** | **Het. pval** | **Ple.pval** | **MR-PRESSO** |  |
| --- | --- | --- | --- | --- | --- | --- | --- | --- |
| **chemokines** | | | | | | | |  |
| CTACK | 11 | 0.054/0.101 | 1.055(0.866,1.286) | 0.595 | 0.837 | 0.644 | 0.841 |  |
| Eotaxin | 11 | -0.081/0.080 | 0.922(0.789,1.078) | 0.310 | 0.181 | 0.453 | 0.192 |  |
| GROa | 11 | -0.115/0.103 | 0.891(0.729,1.090) | 0.262 | 0.893 | 0.749 | 0.890 |  |
| IP10 | 11 | -0.125/0.100 | 0.882(0.725,1.074) | 0.212 | 0.693 | 0.165 | 0.698 |  |
| MCP1 | 11 | -0.057/0.083 | 0.944(0.802,1.111) | 0.490 | 0.122 | 0.080 | 0.137 |  |
| MCP3 | 11 | -0.269/0.185 | 0.764(0.532,1.097) | 0.144 | 0.902 | 0.479 | 0.910 |  |
| MIG | 11 | -0.017/0.100 | 0.983(0.808,1.196) | 0.862 | 0.585 | 0.420 | 0.593 |  |
| MIP1a | 11 | -0.110/0.129 | 0.896(0.696,1.154) | 0.394 | 0.105 | 0.697 | 0.118 |  |
| MIP1b | 11 | 0.042/0.073 | 1.043(0.905,1.203) | 0.559 | 0.305 | 0.436 | 0.310 |  |
| RANTES | 11 | -0.046/0.104 | 0.955(0.778,1.171) | 0.655 | 0.865 | 0.930 | 0.859 |  |
| SDF1a | 11 | -0.082/0.092 | 0.921(0.770,1.102) | 0.369 | 0.064 | 0.727 | 0.070 |  |
| **Growth factors** | | | | | | | |  |
| bNGF | 11 | 0.043/0.115 | 1.044(0.834,1.307) | 0.708 | 0.255 | 0.751 | 0.257 |  |
| FGFbasic | 11 | -0.012/0.094 | 0.988(0.822,1.188) | 0.898 | 0.054 | 0.356 | 0.061 |  |
| GCSF | 11 | -0.014/0.078 | 0.986(0.847,1.149) | 0.860 | 0.229 | 0.198 | 0.242 |  |
| HGF | 11 | 0.011/0.076 | 1.011(0.871,1.173) | 0.887 | 0.233 | 0.866 | 0.235 |  |
| MCSF | 11 | -0.202/0.156 | 0.817(0.602,1.110) | 0.197 | 0.091 | 0.473 | 0.102 |  |
| PDGFbb | 11 | -0.063/0.099 | 0.939(0.773,1.141) | 0.526 | 0.016 | 0.880 | 0.021 |  |
| SCF | 11 | -0.049/0.067 | 0.953(0.835,1.087) | 0.470 | 0.437 | 0.576 | 0.438 |  |
| SCGFb | 11 | 0.005/0.100 | 1.005(0.826,1.223) | 0.962 | 0.763 | 0.472 | 0.785 |  |
| VEGF | 11 | -0.095/0.088 | 0.909(0.765,1.081) | 0.280 | 0.142 | 0.364 | 0.154 |  |
| **Interleukins** | | | | | | | |  |
| IL10 | 11 | -0.073/0.123 | 0.930(0.731,1.182) | 0.551 | 0.001 | 0.699 | 0.001 |  |
| IL12p70 | 11 | -0.098/0.116 | 0.907(0.722,1.139) | 0.399 | 0.001 | 0.497 | 0.002 |  |
| IL13 | 11 | -0.185/0.115 | 0.831(0.663,1.042) | 0.109 | 0.238 | 0.365 | 0.264 |  |
| IL16 | 11 | -0.001/0.103 | 1.000(0.817,1.224） | 0.999 | 0.910 | 0.674 | 0.913 |  |
| IL17 | 11 | -0.071/0.095 | 0.931(0.774,1.121) | 0.452 | 0.047 | 0.823 | 0.057 |  |
| IL18 | 11 | -0.089/0.101 | 0.915(0.750,1.115) | 0.378 | 0.908 | 0.326 | 0.913 |  |
| IL1b | 11 | 0.037/0.106 | 1.037(0.843,1.277) | 0.730 | 0.452 | 0.412 | 0.466 |  |
| IL1ra | 11 | -0.078/0.162 | 0.925(0.673,1.271) | 0.631 | 0.004 | 0.960 | 0.005 |  |
| IL2 | 11 | -0.120/0.146 | 0.887(0.667,1.180) | 0.411 | 0.031 | 0.646 | 0.037 |  |
| IL2ra | 11 | -0.042/0.100 | 0.959(0.788,1.167) | 0.676 | 0.506 | 0.927 | 0.514 |  |
| IL4 | 11 | -0.065/0.102 | 0.937(0.767,1.144) | 0.522 | 0.012 | 0.640 | 0.016 |  |
| IL5 | 11 | -0.148/0.116 | 0.862(0.686,1.083) | 0.202 | 0.263 | 0.138 | 0.275 |  |
| IL6 | 11 | -0.023/0.110 | 0.977(0.788,1.211) | 0.831 | 0.003 | 0.653 | 0.004 |  |
| IL7 | 11 | -0.119/0.113 | 0.887(0.711,1.107) | 0.290 | 0.302 | 0.943 | 0.328 |  |
| IL8 | 11 | -0.075/0.106 | 0.927(0.753,1.142) | 0.478 | 0.376 | 0.125 | 0.385 |  |
| IL9 | 11 | -0.090/0.126 | 0.913(0.714,1.169) | 0.472 | 0.115 | 0.276 | 0.130 |  |
| **Others** | | | | | | | |  |
| IFNγ | 11 | -0.041/0.113 | 0.959(0.768,1.198) | 0.715 | 0.003 | 0.724 | 0.004 |  |
| MIF | 11 | 0.045/0.103 | 1.046(0.855,1.280) | 0.663 | 0.624 | 0.534 | 0.640 |  |
| TNFa | 11 | -0.068/0.124 | 0.934(0.732,1.192) | 0.583 | 0.154 | 0.501 | 0.167 |  |
| TNFb | 11 | -0.155/0.158 | 0.856(0.628,1.168) | 0.327 | 0.396 | 0.060 | 0.439 |  |
| TRAIL | 11 | -0.054/0.067 | 0.947(0.830,1.080) | 0.418 | 0.481 | 0.798 | 0.481 |  |

Supplementary Table 3

Forest plots of the causal relationship between generalized epilepsy and 41 inflammatory factors in the result of IVW in the reverse MR analysis
